# Upregulation of cystathionine-*β*-synthetase expression contributes to inflammatory pain in rat temporomandibular joint

**DOI:** 10.1186/1744-8069-10-9

**Published:** 2014-02-03

**Authors:** Xiuhua Miao, Xiaowen Meng, Geping Wu, Zhong Ju, Hong-Hong Zhang, Shufen Hu, Guang-Yin Xu

**Affiliations:** 1 The Affiliated Zhangjiagang Hospital of Soochow University, Zhangjiagang 215600, P.R. China; 2 Laboratory for Translational Pain Medicine, Department of Neurobiology, Jiangsu Key Laboratory of Translational Research and Therapy for Neuro-Psycho-Diseases, Institute of Neuroscience, Soochow University, Suzhou 215123, P.R. China

**Keywords:** Temporomandibular joint inflammation, Pain, Hydrogen sulfide, Cystathionine-β-synthetase, Trigeminal ganglion, Potassium currents

## Abstract

**Background:**

Hydrogen sulfide (H_2_S), an endogenous gaseotransmitter/modulator, is becoming appreciated that it may be involved in a wide variety of processes including inflammation and nociception. However, the role for H_2_S in nociceptive processing in trigeminal ganglion (TG) neuron remains unknown. The aim of this study was designed to investigate whether endogenous H_2_S synthesizing enzyme cystathionine-β-synthetase (CBS) plays a role in inflammatory pain in temporomandibular joint (TMJ).

**Methods:**

TMJ inflammatory pain was induced by injection of complete Freund’s adjuvant (CFA) into TMJ of adult male rats. Von Frey filaments were used to examine pain behavioral responses in rats following injection of CFA or normal saline (NS). Whole cell patch clamp recordings were employed on acutely isolated TG neurons from rats 2 days after CFA injection. Western blot analysis was carried out to measure protein expression in TGs.

**Results:**

Injection of CFA into TMJ produced a time dependent hyperalgesia as evidenced by reduced escape threshold in rats responding to VFF stimulation. The reduced escape threshold was partially reversed by injection of *O*-(Carboxymethyl) hydroxylamine hemihydrochloride (AOAA), an inhibitor for CBS, in a dose-dependent manner. CFA injection led to a marked upregulation of CBS expression when compared with age-matched controls. CFA injection enhanced neuronal excitability as evidenced by depolarization of resting membrane potentials, reduction in rheobase, and an increase in number of action potentials evoked by 2 and 3 times rheobase current stimulation and by a ramp current stimulation of TG neurons innervating the TMJ area. CFA injection also led to a reduction of *I*_K_ but not *I*_A_ current density of TG neurons. Application of AOAA in TMJ area reduced the production of H_2_S in TGs and reversed the enhanced neural hyperexcitability and increased the *I*_K_ currents of TG neurons.

**Conclusion:**

These data together with our previous report indicate that endogenous H_2_S generating enzyme CBS plays an important role in TMJ inflammation, which is likely mediated by inhibition of I_K_ currents, thus identifying a specific molecular mechanism underlying pain and sensitization in TMJ inflammation.

## Introduction

Hydrogen sulfide (H_2_S), a gas synthesized by the endogenous enzymes cystathionine-β-synthetase (CBS) and cystathionine-γ-lyase (CSE), is increasingly recognized as a biologically important signaling molecule in various tissues and pathophysiological processes including pain and inflammation [1-7]. Its putative role as a neurotransmitter is supported by recent reports on its effects on hippocampal neurons as well as peripheral sensory neurons [7-9]. With respect to the latter, intraplantar injection of NaHS (a commonly used H_2_S donor) in rat hindpaws produces mechanical hyperalgesia [8]. H_2_S generation is enhanced in formalin [9] and carrageenan [10] model of persistent inflammatory pain. Colonic administration of H_2_S enhances pain behaviors in response to CRD in mice [3] and rats [11]. Although there is a discrepancy in the processing of nociceptive signaling [12], a growing body of evidence suggests that H_2_S plays an important effect on primary sensory neurons innervating somatic and visceral organs [8,13,14]. However, the role of H_2_S on trigeminal ganglion (TG) neurons under pathophysiological conditions remains unknown.

We have previously demonstrated that the endogenous H_2_S producing enzyme cystathionine-β-synthetase (CBS) was abundantly expressed in rat TG neurons [15]. Application of H_2_S donor NaHS enhanced excitability and suppressed the voltage-gated *I*_K_ of TG neurons *in vitro* and reduced escape threshold of in healthy rats [15]. These findings suggest that CBS-H_2_S signaling pathway plays an important role in nociceptive pathway in TG under physiological conditions. However, whether CBS-H_2_S signaling pathway plays a role in TG neurons under pathophysiological conditions is unclear. The aims of the present study were therefore to determine roles of the endogenous H_2_S synthyzing enzyme CBS in TGs in rats with TMJ inflammation. We hypothesized that TMJ inflammation-induced hyperalgesia is mediated by upregulation of *cbs* gene expression and that activation of CBS-H_2_S signaling enhances neuronal excitability via suppression of potassium currents of TMJ-projecting TG neurons, thus contributing to hyperalgesia in TMJ after inflammation. Since we have showed that NaHS suppress the *I*_K_ current density in our previous study, therefore, in the present study, we further investigated the role of voltage-gated K channels in TMJ inflammation. To test this hypothesis, we examined cbs gene expression and determined neuronal excitability and potassium current densities of TMJ-projecting TG neurons in an experimental rat model of TMJ inflammation induced by CFA injection. We demonstrated an upregulation of CBS expression, enhanced neuronal excitability, and an inhibition of sustained potassium current (*I*_K_) density of TG neurons after CFA injection. Administration of a CBS inhibitor reversed hyperexcitability, increased *I*_K_ current density of TG neurons, and attenuated pain responses. These observations support a pro-nociceptive role for H_2_S in TMJ inflammatory pain.

## Methods and materials

### Animals

Experiments were performed on adult male Sprague-Dawley rats (220 ± 20 g). Animals were housed under controlled conditions (07:00 ~ 19:00, lighting, 24 ± 2°C) with free access to a standard laboratory diet and fresh water. Care and handling of these animals were approved by the Institutional Animal Care and Use Committee of Soochow University and were in accordance with the guidelines of the International Association for the Study of Pain.

### DiI labeling

Temporomandibular joint receptive field specific TG neurons were labeled by injection of 1, 19-dioleyl-3, 3, 39, 3-tetramethy-lindocarbocyanine methanesulfonate (DiI; Invitrogen, Carlsbad, California) as described previous [16]. The skin overlying the TMJ was shaved. The injection site was identified by palpating the zygomatic arch and mandible. DiI (25 mg in 0.5 ml methanol) was slowly injected into TMJ (1 μl/site, 3 sites in each side). Multiple small injections of the tracers were made to limit the spread of the tracer into untargeted tissues [17]. To prevent leakage, needle was left in place for ~2 min for each injection. Ten days later, the TGs were dissected out for patch clamp recordings. The appearance of the injected tracer in neurons indicates their innervation zone in the overlying skin.

### Induction of TMJ inflammation

To induce TMJ inflammation, Complete Freund’s Adjuvant (CFA) (50 μl, 1:1 oil/saline suspension, Sigma, St. Louis, MO) was injected into the right side of the TMJ capsule, as described in previous studies [16,18,19]. For control rats, 50 μl of normal saline (NS) was injected into the TMJ capsule. To prevent leakage, CFA or NS was injected slowly over a time span of 2 min and the needle was left in place for ~2 min.

### Mechanical threshold for escape behavior

On the day of testing, rats were weighed and virissas were carefully shaved. The mechanical threshold for escape behavior of ipsilateral and contralateral facial skin regions were tested as described previously [15]. The mechanical stimulation was applied to the skin above the inflamed TMJ. In brief, rats were first placed individually in small plastic cages and were allowed to adapt to the observation cage and testing environment for ~1 hr. During this period, the experimenter slowly reached into the cage to touch the walls of the cage with a plastic rod. After the rats were habituated to the reaching movements, the series of mechanical stimulations were started. The mechanical response threshold of escape behavior was measured in control and inflamed rats. A graded series of von Frey filaments were used. The filaments produced a bending force of 0.55, 0.93, 1.61, 1.98, 2.74, 4.87, 7.37, 11.42, 15.76, 20.30, and 38.69 g. A descending series of the filaments were used when the rat responded to the starting filament. Each filament was tested five times at an interval of a few seconds. If head withdrawal was observed at least three times after probing with a filament, the rat was considered responsive to that filament. The response threshold was defined as the lowest force of the filaments that produced at least three withdrawal responses in five tests. The response was observed to belong to one or more of the following responses: 1) The rat slowly turns the head away or briskly moves it backward when the stimulation is applied, and sometimes a single face wipe ipsilateral to the stimulated area occurs; 2) The rat avoids further contact with the stimulus object, either passively by moving its body away from the stimulating object to assume a crouching position against the cage wall, or actively by attacking the stimulus object, making biting and grabbing movements; 3) The rat displays an uninterrupted series of at least three face-wash strokes directed toward the stimulated facial area [15,20].

### Dissociation of TG neurons

Isolation of TG neurons from adult male rats has been described previously [21,22]. Briefly, animals 10 days after injection of DiI were killed by cervical dislocation, followed by decapitation. The TGs were then bilaterally dissected out and transferred to an ice-cold, oxygenated fresh dissecting solution, which contained (in mM): 130 NaCl, 5 KCl, 2 KH_2_PO_4_, 1.5 CaCl_2_, 6 MgSO_4_, 10 glucose, and 10 HEPES, pH 7.2 (osmolarity: 305 mOsm). After removal of connective tissue, ganglia were transferred to 5 ml of dissecting solution containing collagenase D (1.8–2.0 mg/ml, Roche; Indianapolis, IN) and trypsin (1.2 mg/ml, Sigma; St. Louis, MO) and incubated for 1.5 hrs at 34.5°C. TGs were then taken from the enzyme solution, washed, and transferred to 2 ml of the dissecting solution containing DNase (0.5 mg/ml, Sigma, St. Louis, MO). A single-cell suspension was subsequently obtained by repeated trituration through flame-polished glass pipettes. Cells were plated onto acid-cleaned glass coverslips.

### Patch-clamp recordings

As described previously [15], coverslips containing adherent TG cells were put in a small recording chamber (0.5 ml volume) and attached to the stage of an inverting microscope (Olympus, Japan). For patch-clamp recording experiments, cells were continuously superfused (1.5 ml/min) at room temperature with normal external solution containing (in mM) 130 NaCl, 5 KCl, 2 KH_2_PO_4_, 2.5 CaCl_2_, 1 MgCl_2_, 10 HEPES, and 10 glucose, with pH adjusted to 7.4 with NaOH (osmolarity: 295-300 mOsm). DiI-labeled neurons were identified by the bright red fluorescence in the cytoplasm. Recording pipettes were pulled from borosilicate glass tubing using a horizontal puller (P-97, Sutter Instruments) and typically had a resistance of 3.5-4.5MΩ when filled with normal external solution before being used immediately to obtain a gigaohm seal. Tip potential was zeroed before membrane-pipette seals were formed. The voltage was clamped at -60 mV by an EPC10 amplifier (HEKA, Germany). Capacitive transients were corrected using capacitive cancellation circuitry on the amplifier that yielded the whole cell capacitance and access resistance. Up to 80% of the series resistance was compensated electronically. Considering the peak outward current amplitudes of 10 nA, the estimated voltage errors from the uncompensated series resistance would be 10 mV. The leak currents at -60 mV were always below 20 pA and were not corrected. The action potentials (APs) were filtered at 2–5 kHz and sampled at 50 or 100 μs/point. Data were acquired and stored on a computer for later analysis using Patch Master (HEKA, Germany). All experiments were performed at room temperature (~22°C). Only neurons with a stable initial resting potential, which drifted by less than 2–3 mV during the 10 min of baseline recording, were used in these experiments. Cells were characterized by their resting membrane potentials, input resistances (Rm) and cell capacitances. Stimulating ramps of linearly increasing current (range 0.1, 0.3 and 0.5 nA/s) were chosen to produce more APs over a 1-second depolarization for each tested neuron. In addition to the number of APs during the ramp, the AP threshold, AP amplitude and duration elicited by current stimulation were analyzed in this study as described previously [15].

### Isolation of voltage-gated potassium (K_V_) currents

To record K_V_ currents, Na^+^ in control external solution was replaced with equimolar choline and Ca^2+^ concentration was reduced to 0.03 mM to suppress Ca^2+^ currents and to prevent Ca^2+^ channels becoming Na^+^ conducting. The reduced external Ca^2+^ would also be expected to suppress Ca^2+^-activated K^+^ current [23]. The following two kinetically distinct Kv currents were isolated by the biophysical analysis and pharmacological approaches described in previous studies: *I*_A_ and *I*_K_[15,24,25]. *I*_A_ and *I*_K_ were separated biophysically by manipulating the holding potentials. For total voltage-gated potassium current (*I*_Total_), the membrane potential was held at -100 mV and voltage steps were from -50 to +90 mV with10-mV increments and 400 ms duration. For sustained voltage-gated potassium current (*I*_K_), the membrane potential was held at -50 mV and the voltage steps were the same as above. Subtraction of *I*_K_ from *I*_Total_ represented *I*_A_. To control for changes in cell size, the current density (pA/pF) was measured by dividing the current amplitude by whole cell membrane capacitance, which was obtained by reading the value for whole cell input capacitance cancellation directly from the patch-clamp amplifier.

### Western blotting

Trigeminal ganglion from CFA-treated rats (2 days) or age-matched control rats were dissected out and lyzed in 120 μl of radioimmunoprecipitation assay buffer containing 1% NP-40, 0.5% Na deoxycholate, 0.1% SDS, PMSF (10 μl/ml), and aprotinin (30 μl/ml; Sigma). The cell lysates were then microfuged at 15,000 rpm for 30 min at 4°C. The concentration of protein in homogenate was determined using a BCA reagent (Beyotime, CHN). Twenty micrograms (20 μg) of proteins for CBS expression were loaded onto a 10% Tris-HCl SDS-PAGE gel (Bio-Rad, Hercules, CA). After electrophoresis, the proteins were electrotransferred onto polyvinyldifluoride membrane (Millipore) at 200 mA for 2 hrs at 4°C. The membrane was incubated in 25 ml of blocking buffer (1XTBS with 5% w/v fat-free dry milk) for 2 hrs at room temperature. The membrane was then incubated with the primary antibodies for overnight at 4°C. Primary antibodies used were mouse anti-CBS (1:1000; Abnova), mouse anti-CSE (1:1000, Abnova) and mouse anti-actin (1:1000; Chemicon, Temecula, CA). After incubation, the membrane was washed with TBST (1XTBS and 5% Tween 20) three times for 15 min each and incubated with anti-mouse peroxidase-conjugated secondary antibody (1:4000; Chemicon) for 2 hrs at room temperature. The membrane was then washed with TBST three times for 15 min each. The immunoreactive proteins were detected by enhanced chemiluminescence (ECL kit; Amersham Biosciences, Arlington Heights, IL). The bands recognized by primary antibodies were visualized by exposure of the membrane onto an x-ray film. All samples were normalized to β-actin as control. For quantification of CBS or CSE protein levels, the photographs were digitalized and analyzed using a scanner (Bio-Rad imaging system Bio-Rad GelDoc XRS^+^).

### Real-time PCR for CBS mRNA

Purification of total RNA from TG tissues was performed with RNeasy Mini Kits (Qiagen, Valencia, CA, USA) according to the manufacturer’s instructions. RNA purity and concentration were determined spectrophotometrically. RNA was only used if the ratio between spectrophotometer readings (260 nm: 280 nm) was between 1.8 and 2.0. A reverse transcription and first strand cDNA synthesis was performed using an Omniscript RT kit (QIAGEN) (Invitrogen) following the supplier’s instructions. For detecting mRNA level of *cbs*, real time PCR was conducted on an ABI 7500 Fluorescent Quantitative PCR system (Applied Biosystems, Bedford, MA, USA). A 25 μL reaction mixture contained 1 μL of cDNA from samples, 12.5 μL of 2 × SYBR Green qPCR Master Mix, 1 μL primers (10 mM), and 10.5 μL of RNase/DNase free water. Amplification conditions involved a pre-incubation at 95°C for 1 min followed by amplification of the target DNA for 40 cycles (95°C for 10 s and 60°C for 20 s), and the fluorescence collection at 60°C. Melting curve analysis was performed at a linear temperature transition rate of 0.5°C/s from 60°C to 95°C with continuous fluorescence acquisition. Relative expression level for *cbs* gene was normalized by the Ct value of β-actin (internal control) using a 2^-ΔΔ^Ct relative quantification method. The sequences of the primers for *cbs* were 5’-GAACCAGACGGAGCAAACAG-3′ (forward) and 5′-GGCGAAGGAATCGTCATCA-3′ (reverse), giving a 121-bp amplicon. All experiments were repeated three times for reproducibility.

### Measurement of hydrogen sulfide (H_2_S) concentration

H_2_S level was measured using a previously described method [26,27]. Briefly, trigeminal ganglion tissues were homogenized in 250 μl of ice-cold 100 mM potassiumphosphate buffer (pH = 7.4) containing trichloroacetic acid (10% w/v). Zinc acetate (1% w/v, 250 μl) was injected to trap the generated H_2_S. A solution of N,N-dimethyl-p-phenylenediamine sulfate (20 μM; 133 μl) in 7.2 M HCl and FeCl_3_ (30 μM; 133 μl) in 1.2 M HCl was added. Absorbance at 670 nm of the resulting mixture (250 μl) was determined after 10 min using a 96-well microplate reader (Bio-Rad). The H_2_S concentration of each sample was calculated against a calibration curve of NaSH (0–250 μM) and results were expressed as nmol/mg proteins.

### Drug application

*O*-(Carboxymethyl) hydroxylamine hemihydrochloride (AOAA) and L-cysteine (L-Cys) were purchased from Sigma–Aldrich (St. Louis, MO) and was freshly prepared in normal external solution. AOAA or L-Cys or an equal volume of normal saline (NS) used as control was injected subcutaneously into the TMJ area. For behavioral studies, AOAA at different doses (3, 6 and 9 mg/kg body weight) was injected once 2 days after CFA injection. L-Cys at different doses (50, 500 and 1000 μM in 100 μl) was injected into TMJ area in healthy control rats. Escape threshold was determined after injection of AOAA or L-Cys. For patch clamp experiments, AOAA at 9 mg/kg body weight was injected 8 hrs after CFA injection three times a day for consecutive 2 days. Dissection of TGs was performed 30 minutes after last injection of AOAA or NS.

### Rotarod test

Rotarod testing was examined using a previously described method [28]. Briefly, rats were first placed on the rotarod at a given speed (from 5 rpm to 15 rpm) 1 day or 2 consecutive days for training before the beginning of the experiment. After this training most rats step voluntarily from the operator’s hand onto the rod. The length of time that each rat is able to stay on the rod at a given rotation speed (15 rpm) was recorded before and after administration of AOAA.

### Data analysis

All values are given as mean±SEM. No neuron with a resting membrane potential more depolarized than -40 mV was included in the data analysis. Statistical analyses were conducted using commercial software OriginPro 8 (OriginLab, US) and Matlab (Mathworks, US). Normality was checked for all data before analyses. Statistical significance was determined by two sample t-Test, Mann-Whitney test, Dunn’s post hoc test following Friedman ANOVA, Kruskal-Wallis ANOVA followed by Tukey post hoc test, as appropriate. P < 0.05 was considered statistically significant.

## Results

### CBS inhibitor AOAA attenuates inflammatory hyperalgesia

**Figure 1:**
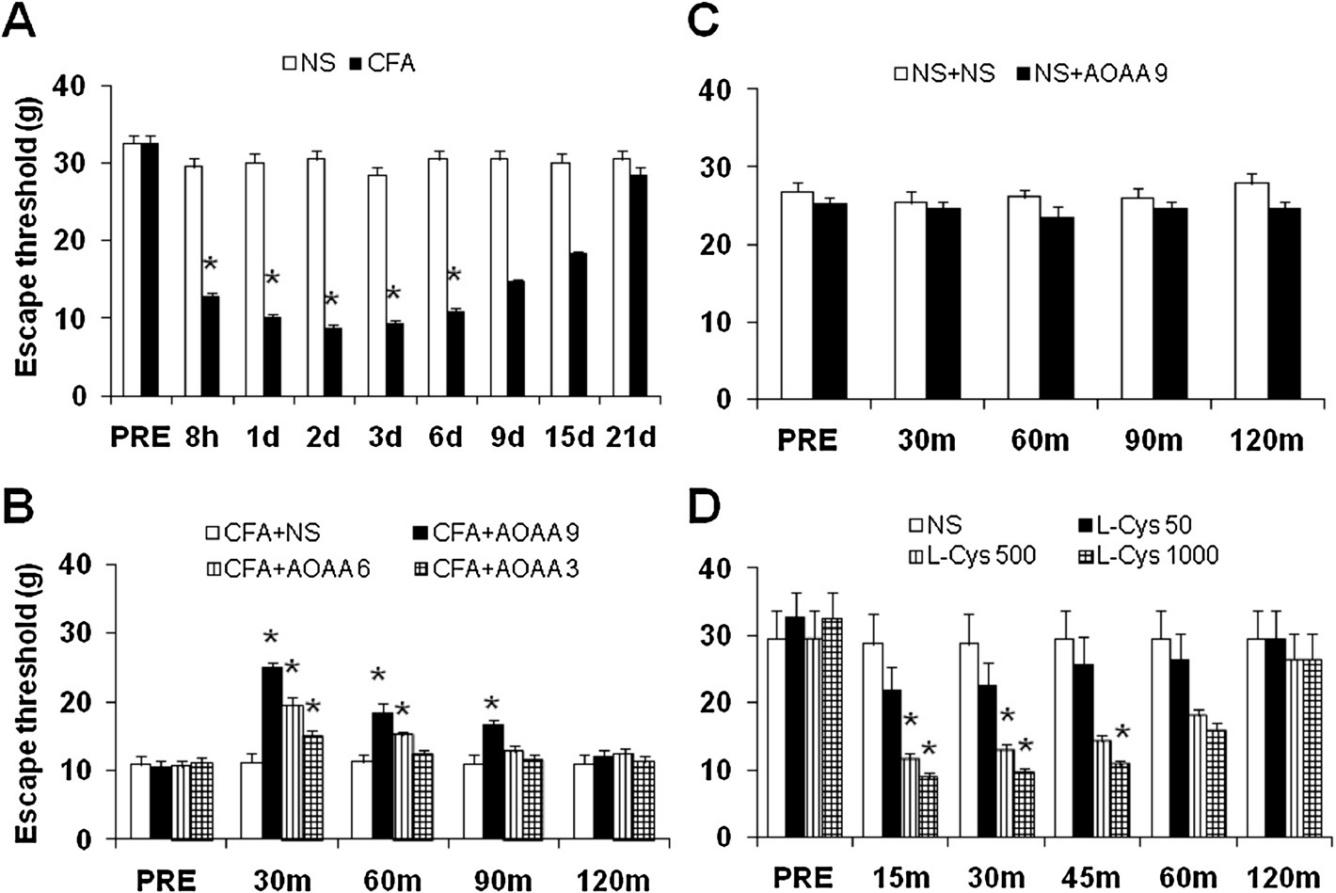
**CBS inhibitor attenuates mechanical hyperalgesia in CFA rats.** Escape threshold (ET) was determined by measuring mechanical threshold in response to von Frey filaments. **(A)** Injection of CFA into TMJ area of rats (n = 9) significantly decreased ET (filled bars) while injection of the same volume of normal saline (NS, n = 9) did not alter ET (open bars) significantly. The lowered ET started 8 hours after CFA injection and lasted for ~6 days (n = 9, *p < 0.05, compared with that before CFA injection (PRE), Dunn’s post hoc test following Friedman ANOVA). **(B)** Subcutaneous injection of AOAA 8 hours after CFA injection had a significant effect on the ET in CFA rats, in a dose- and time-dependent manner. ET was increased 30 minutes after administration of AOAA in CFA rats in a dose dependent manner (n = 13 rats for each group, *p < 0.05 compared with NS, Kruskal-Wallis ANOVA followed by Tukey post hoc test). **(C)** Subcutaneous injection of AOAA at 9 mg/kg body weight or same volume of NS had no significant effects on ET in healthy control rats (n = 13 rats for each group). **(D)** Subcutaneous injection of L-Cysteine (L-Cys), an endogenous substrate for CBS to generate H_2_S, produced mechanical hyperalgesia in healthy control rats, in a dose- and time-dependent manner (n = 6 rats for each group, *p < 0.05 compared with NS, Kruskal-Wallis ANOVA followed by Tukey post hoc test).

Escape threshold (ET) was determined by measuring mechanical threshold in response to von Frey filaments. The ET was significantly lower after CFA injection than those before CFA injection (PRE). The lowered ET started 8 hours after CFA injection and lasted for ~6 days, indicating an establishment of mechanical hyperalgesia in rats. The ET was returned to normal level 9 days after CFA injection (Figure 1A, n = 9, *p < 0.05 compared with before CFA injection, Dunn’s post hoc test following Friedman ANOVA). In contrast, injection of the same volume of normal saline (NS) into TMJ area did not significantly alter the ET in rats (Figure 1A, n = 9, Dunn’s post hoc test following Friedman ANOVA).

To determine whether the endogenous H_2_S producing enzyme CBS are involved in CFA-induced mechanical hypersensitivity, AOAA, a potent CBS inhibitor, was administrated subcutaneously in TMJ. Injection of AOAA had a significant effect on ET in CFA rats (Figure 1B). ET was increased 30 minutes after administration of AOAA in CFA rats, in a dose dependent manner (Figure 1B, n = 13 rats for each group; *p < 0.05 compared with NS, Kruskal-Wallis ANOVA followed by Tukey post hoc test). Three doses of AOAA (3, 6, 9 mg/kg body weight) were used in this study. The optimized dose for AOAA to produce the maximal effect was 9 mg/kg body weight. We then determined the time course of AOAA effects. The effect of AOAA at 3, 6 and 9 mg/kg lasted ~30, 60 and 90 min, respectively (Figure 1B, n = 13 rats for each group; *p < 0.05 compared with NS, Kruskal-Wallis ANOVA followed by Tukey post hoc test). These results suggest that inhibition of H_2_S production attenuated mechanical hyperalgesia in rats with TMJ inflammation. To further confirm the effect of AOAA in CFA rats, AOAA was administrated in age-matched healthy control rats. AOAA at 9 mg/kg or NS had no significant effects on the ET in healthy control rats (Figure 1C, n = 13 rats for each group), suggesting that this agent did not act as a non-specific analgesic and that CBS do not normally participate in the responses to mechanic stimulation in normal conditions.

If H_2_S generated endogenously contribute to the development of mechanical hyperalgesia in CFA-injected animals, the exogenous H_2_S would expect to produce hyperalgesia in healthy rats. This is supported by our previous report that administration of H_2_S donor NaHS produced mechanical hyperalgesia [15]. To further ascertain H_2_S effect, we administered L-Cys, an endogenous substrate for CBS to generate H_2_S, in healthy rats in present study. Similar to NaHS [15], L-Cys produced mechanical hyperalgesia in a dose-dependent manner (Figure 1D, n = 6 rats for each group, *p < 0.05 compared with NS, Kruskal-Wallis ANOVA followed by Tukey post hoc test). The hyperalgesic effect of L-Cys persisted for ~45 min. These data demonstrate that H_2_S produces an acute hyperalgesic effect in healthy rats, which partially mimics the effect induced by CFA injection.

### CFA injection increases expression of CBS in TG

**Figure 2:**
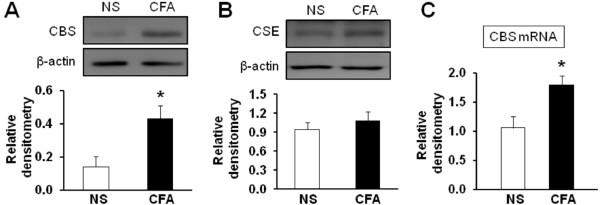
**CFA injection enhances expression of CBS in TG. (A)** CBS protein expression was greatly enhanced in CFA-injected rats when compared with age- and sex-matched controls (NS, n = 4; CFA, n = 4, *p < 0.05, compared with NS, two sample t-Test). **(B)** CSE protein expression was not significantly altered in CFA-injected rats when compared with controls. **(C)** RT-PCR assays demonstrated a significant upregulation of CBS mRNA expression in TGs in CFA-injected rats (NS, n = 4; CFA, n = 4, *p < 0.05, compared with NS, two sample t-Test).

To determine whether CFA injection upregulated CBS expression, TGs were dissected out 2 days after CFA or NS injection. The reason why selected this time point to perform experiments is because the escape threshold at this point is at bottom of the time curve (Figure 1A) and also to minimize the suffering from pain. As shown in Figure 2A, CFA injection dramatically increased CBS expression in TGs (*p < 0.05 compared with NS, n = 4 for each group, two sample t-Test). We also examined expression of cystathionine-γ-lyase (CSE), another endogenous H_2_S producing enzyme. Expression of CSE was not altered significantly 2 days after CFA injection when compared with controls (Figure 2B). To determine whether CFA injection altered CBS expression at gene level, the expression of CBS mRNA was examined 2 days after CFA injection. CFA injection markedly enhanced CBS mRNA level when compared with NS group (Figure 2C, *p < 0.05, n = 4 for each group, two sample t-Test).

### CFA injection enhances excitability of TMJ neurons

**Figure 3:**
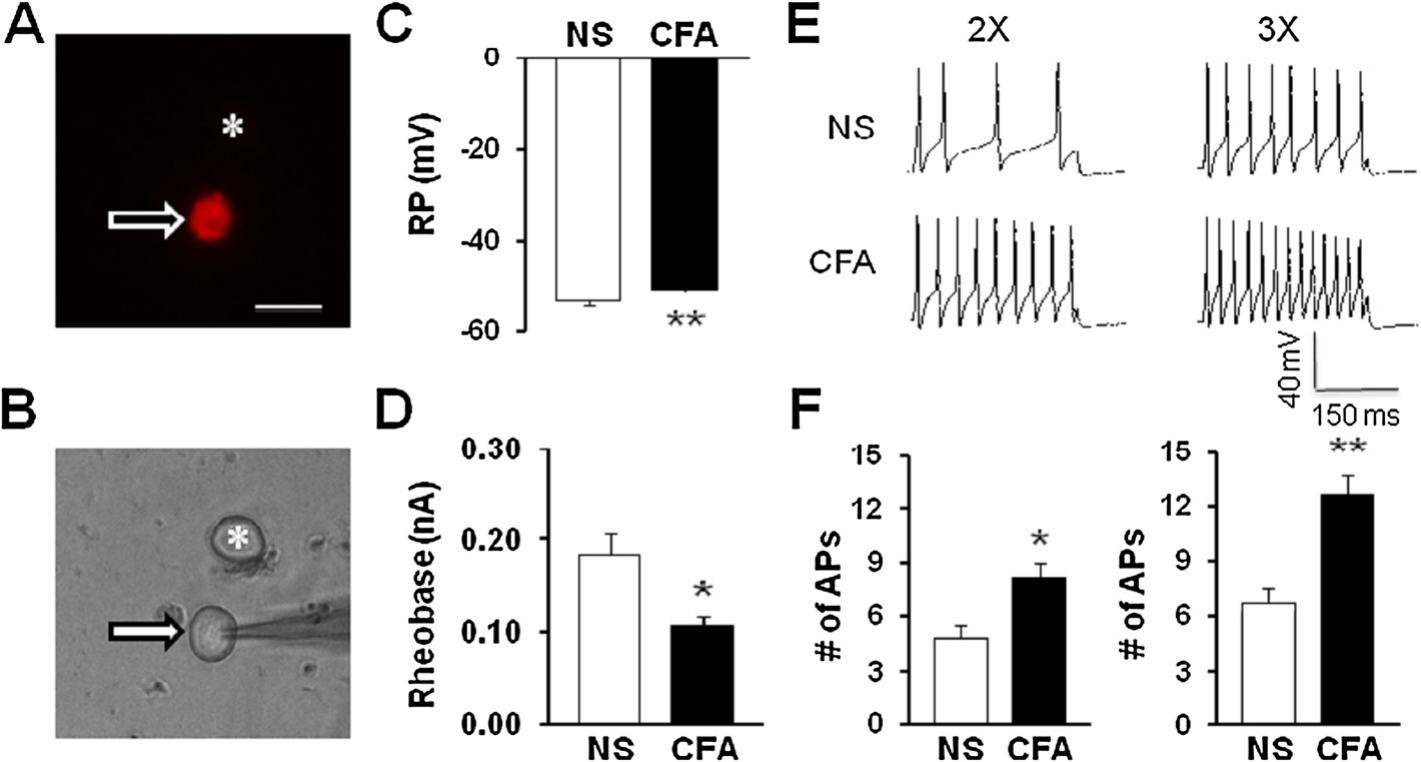
**CFA injection increases excitability of TMJ neurons. (A)** An example of a Dil-labeled TG neuron (arrow). A star showing the place where a neuron is not labeled by DiI. **(B)** A phase image of the same TG neuron labeled by DiI is shown on the bottom (arrow) and the neuron not labeled by DiI is shown on the top (*). Bar = 50 μm. Patch clamp recordings were performed on DiI labeled TG neurons. A total of 22 DiI-labeled neurons from control rats and 26 DiI-labeled neurons from CFA-treated rats were recorded under current clamp conditions. **(C)** CFA injection significantly depolarized resting membrane potentials (RP) of TG neurons innervating TMJ (**p < 0.01, compared with NS, Mann-Whitney test). **(D)** CFA injection markedly reduced rheobase (*p < 0.05, compared with NS, Mann-Whitney test). **(E)** Example of action potential (AP) trances evoked by 2X (left) and 3X (right) rheobase current stimulation of TMJ innervating TG neurons from NS- (top) and CFA- (bottom) injected rats. **(F)** Bar graphs showed that CFA injection greatly increased numbers of AP evoked by 2X (left) and 3X (right) rheobase current stimulation (*p < 0.05, **p < 0.01, compared with NS, Mann-Whitney test).

To determine whether CFA injection altered neuronal excitability, we next investigated intrinsic membrane properties including resting membrane potentials (RP), current threshold (rheobase), and pattern of firings in response to depolarizing current stimulation of TG neurons innervating the TMJ. TMJ innervating TG neuron, labeled by DiI (Figure 3A), were identified under microscope (Figure 3B). The average diameter was 25.9 ± 0.75 μm (n = 22 cells) for control rats and 24.6 ± 5.39 μm (n = 26 cells) for CFA rats (Table 1). The resting membrane potentials of DiI labeled TG neurons were significantly altered after CFA injection (Figure 3C, **p < 0.01, compared with NS, Normality test following Mann-Whitney test). The average RP were -54.23±1.07 mV (n = 22) and -50.69±0.81 mV (n = 26) for control and CFA rats, respectively. Rheobase, the minimal stimulation current to evoke one action potential (AP), was also determined. The average rheobase of TMJ neurons was 0.18±0.02 nA (n = 22) and 0.11±0.01 nA (n = 26) for control and CFA rats, respectively. CFA injection led to a marked reduction in rheobase when compared with that of NS injection (Figure 3D, *p *<* 0.05, compared with NS, Mann-Whitney test). In addition, the numbers of APs in response to a current stimulation (2×, 3× rheobase, 300 ms in duration) were examined 2 days after CFA injection. The number of AP numbers in response to 2× and 3× current stimulation in age-matched control rats was 5.32±0.79 (n = 22) and 7.5±1.10 (n = 22), respectively. In CFA injected rats, the number of AP numbers in response to 2× and 3× current stimulation was 8.27±0.78 (n = 26) and 12.58±1.20 (n = 26), respectively (Figure 3E and F, *p *<* 0.05, **p < 0.01, Mann-Whitney test). Figure 3E are representative voltage trace in response to 2× and 3× rheobase current stimulations 2 days after application of NS (top) or CFA (bottom). This increase in spike numbers was not due to a change in cell input resistance because cell input resistance was not altered significantly after CFA injection (NS vs. CFA; 635.5 ± 44.2 MΩ vs. 643.6 ± 21.8 MΩ, p > 0.05, two sample t-Test, Table 1). These results suggest that CFA injection increases neuronal excitability.

**Table 1:** Membrane characteristics of TMJ innervating TG neurons of control (NS) and CFA-injected rats

	**NS (n = 22)**	**CFA (n = 26)**	***p *Value**
**Cell size (μm)**	25.9 ± 0.75	24.6 ± 5.39	NS
**Cm (pF)**	29.16 ± 1.23	26.53 ± 1.09	NS
**RP (mV)**	-54.23 ± 1.07	-50.69 ± 0.81	< 0.01
**Rin (MΩ)**	635.5 ± 44.2	643.6 ± 21.8	NS
**Rheobase (nA)**	0.18 ± 0.02	0.11 ± 0.01	< 0.05
**# of APs (2Xrheobase)**	5.32 ± 0.79	8.27 ± 0.78	< 0.05
**# of APs (3Xrheobase)**	7.5 ± 1.10	12.58 ± 1.20	< 0.01
**AP Threshold (mV)**	-29.33 ± 1.77	-30.68 ± 1.42	NS
**AP Amplitude (mV)**	97.51 ± 2.34	87.35 ± 3.22	<0.05
**AP Overshoot (mV)**	41.24 ± 2.28	35.43 ± 2.84	NS
**AP Duration (ms)**	2.49 ± 0.17	2.05 ± 0.12	NS
**# of APs (Ramp 100 pA)**	1.32 ± 0.43	2.31 ± 0.76	NS
**# of APs (Ramp 300 pA)**	8.19 ± 1.65	15.15 ± 1.76	< 0.01
**# of APs (Ramp 500 pA)**	17.45 ± 2.87	29.15 ± 2.40	< 0.01

Values are mean ± SEM, with sample size in parenthesis; Cm, membrane capacitance; RP, Resting membrane potential; AP, Action potential; Rin, Input resistance; NS, No significance; p values were determined by two sample *t*-Test or Mann-Whitney test where appropriate.

**Figure 4:**
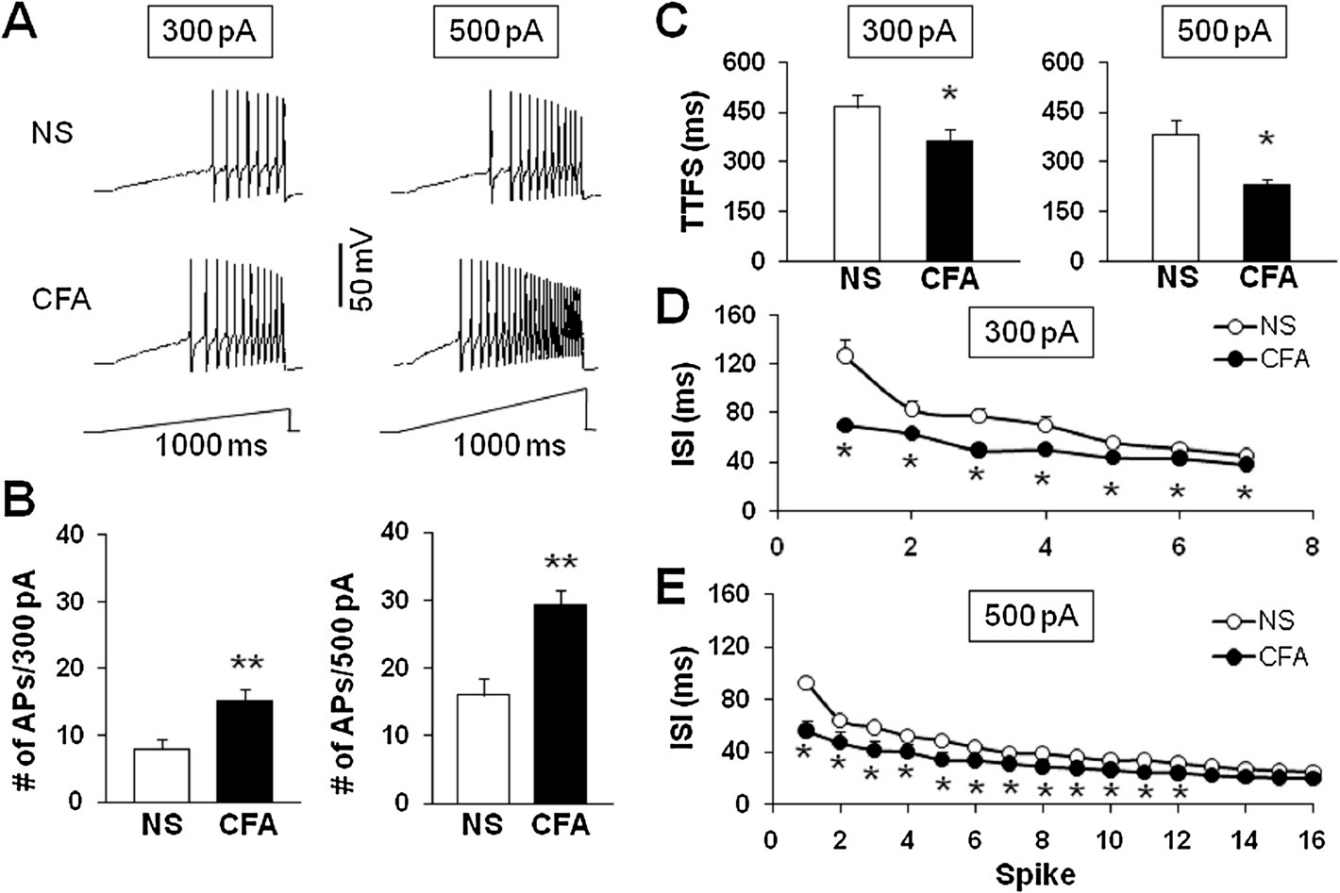
**CFA injection increases the number of action potentials evoked by ramp current stimulation. (A)** Examples of APs evoked by 300 pA (left) and 500 pA (right) ramp current stimulation from NS (top) and CFA-injected (bottom) rats. **(B)** Bar graph showed a significant increase in numbers of APs evoked by 300 pA (left) and 500 pA (right) ramp current stimulation in NS- or CFA-injected rats (**p < 0.01, compared with NS, Mann-Whitney test). **(C)** Bar graph representing the mean time to first spike (TTFS) in response to a 300 pA (left) or 500 pA (right) ramp current injection from NS or CFA-injected rats (*p < 0.05, compared with NS, Mann-Whitney test). **(D)** Mean interspike interval (ISI) in a spike train of TMJ neurons responding to a 300 pA ramp current stimulation from NS- or CFA-injected rats (*p < 0.05 compared with NS, Mann-Whitney test). **(E)** Mean ISI in a spike train of TMJ neurons responding to a 500 pA ramp current stimulation from NS- or CFA-injected rats (*p < 0.05 compared with NS, Mann-Whitney test).

To further compare numbers of AP firing of TG neurons after CFA injection, we also used 1-second ramp current stimulation from 0 to 300 pA or 500 pA (Figure 4). Because APs elicited by ramp current stimulation showed adaptation in some neurons, we counted only overshooting APs (i.e., AP with peak >0 mV). Figure 4A shows the representative voltage traces in response to 300 pA (left) and 500 pA (right) ramp current stimulation 2 days after injection of NS (top) or CFA (bottom). The average numbers of APs in control rats were 8.19±1.65 (n = 22) and 17.45±2.87 (n = 22) for 300 pA and 500 pA, respectively. In CFA injected rats, the average numbers of APs were 15.15±1.76 (n = 26) and 29.15±2.40 (n = 26) for 300 pA and 500 pA, respectively. Injection of CFA significantly increased the number of APs evoked by 300 or 500 pA ramp current injection (Figure 4B). Again, this increase in spike number was not due to a change in cell input resistance because cell input resistance was not altered significantly after CFA injection (NS vs. CFA; 635.5 ± 44.2 MΩ vs. 643.6 ± 21.8 MΩ, p > 0.05, two sample t-Test, Table 1). Furthermore, the time to first spike (TTFS) was significantly decreased by CFA injection (NS vs. CFA: 465.97±35.70 ms vs. 364.22±33.18 ms, *p < 0.05 at the 300 pA depolarization, Figure 4C left; 382.01±45.30 ms vs. 228.56±20.08 ms, *p < 0.05 at the 500 pA depolarization, Figure 4C right). A change in the interspike interval (ISI) in response to a 300 pA and 500 pA current injection was seen both at the beginning of a train of spikes (after 1^th^ spike, NS vs. CFA: 126.48±13.87 ms vs. 69.99±4.66 ms, *p < 0.05 at the 300 pA depolarization, Figure 4D; 92.18±8.06 ms vs. 56.15±2.61 ms, *p < 0.05 at the 500 pA depolarization, Figure 4E) and in the latter parts of the train (after 6^th^ spike, NS vs. CFA: 52.60±3.38 ms vs. 43.11±2.88 ms, *p < 0.05 at the 300 pA depolarization, Mann-Whitney test, Figure 4D; 43.37±4.06 ms vs. 33.49±1.93 ms, *p < 0.05 at the 500 pA depolarization, Mann-Whitney test, Figure 4E), suggesting an effect of CFA injection on spike frequency adaptation of TMJ neurons.

Several additional membrane properties were also examined. AP threshold, AP duration and overshoot, and membrane input resistance were not significantly altered in TMJ projecting neurons from rats after CFA or NS injection (Table 1).

### CFA injection suppresses voltage-gated potassium current of TG neurons

**Figure 5:**
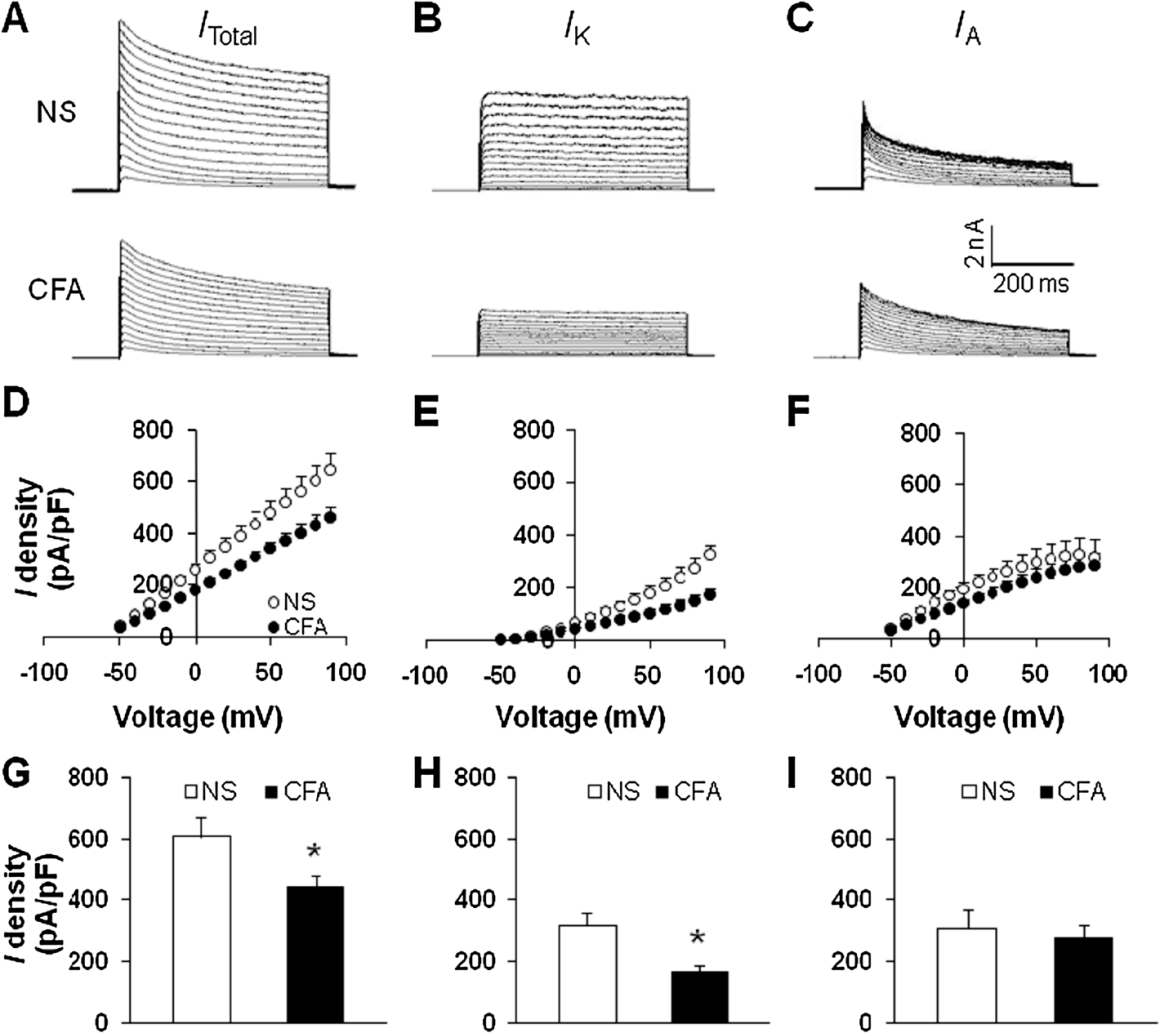
**CFA injection suppresses voltage-gated potassium currents.** Currents were measured at different holding potentials. For total voltage-gated potassium current (I_Total_), the membrane potential was held at -100 mV and voltage steps were from -50 to +90 mV with10-mV increments and 400 ms duration. For sustained voltage-gated potassium current (I_K_), the membrane potential was held at -50 mV and the voltage steps were the same as above. Currents generated by these two protocols were subtracted to produce I_A_. **(A)** Examples of total K_V_ currents recorded from NS (top) and CFA-treated rats (bottom). **(B)** Examples of I_K_ recorded from NS (top) and CFA-treated rats (bottom). **(C)** Examples of I_A_ recorded from NS (top) and CFA-treated rats (bottom). The peak I_Total_**(D)**, I_K_ (E) and I_A_**(F)** versus voltages (I-V) were plotted from cells acutely dissociated from rats treated with NS or CFA. Bar graphs showed the mean peak I_Total_**(G)**, I_K_**(H)**, and I_K_**(I)** densities from NS and CFA-treated rats. The current density (pA/pF) was calculated by dividing the current amplitude by cell membrane capacitance. CFA injection caused a significant reduction of I_Total_ (G, *p < 0.05, compared with NS, two sample t-Test). The I_K_ density was significantly reduced (H, *p < 0.05, compared with NS, two sample t-Test) while the I_A_ was not altered significantly after CFA injection **(I)**.

Because changes in spike frequency and activation thresholds suggest that there was an alteration in voltage-gated potassium (K_V_) channels, we next performed patch-clamp recordings to examine these currents under voltage-clamp conditions. Na^+^ in the control external solution was replaced with equimolar choline and the Ca^2+^ concentration was reduced to 0.03 mM, as described previously [29-31]. A depolarization step from -50 to +90 mV in 10-mV increments with duration of 400 ms activated all K_V_ channels (*I*_Total_; Figure 5A). The peak current-voltage (*I*-V) curves are shown in Figure 5D. However, CFA injection greatly decreased peak current density in DiI-labeled neurons (*p < 0.05, compared with NS, two sample t-Test, Figure 5G). The mean peak current density of total voltage-gated potassium current from NS-treated rats was 644.89 ± 64.58 pA/pF (n = 7), and the mean peak current density of total voltage-gated potassium current from CFA-treated rats was 462.13 ± 37.82 pA/pF (n = 7). Because there were two main types of K_V_ currents (*I*_A_ and *I*_K_) described in nociceptive TG neurons, we then isolated these two kinetically different K_V_ currents by manipulating the holding membrane potential. A depolarization step -50 to +90 mV in 10-mV increments with duration of 400 ms activated most of the sustained K_V_ channels (Figure 5B) but not A-type K_V_ channels. Subtraction of *I*_K_ from *I*_Total_ represented *I*_A_ (Figure 5C). In this experiment, *I*_K_ density was remarkably reduced after CFA application (*p < 0.05, compared with NS, two sample t-Test, Figure 5H). The mean peak current density of *I*_K_ from NS-treated rats was 326.19 ± 37.84 pA/pF (n = 7), and the mean peak current density of *I*_K_ from CFA-treated rats was 173.55 ± 23.08 pA/pF (n = 7). Whereas *I*_A_ density was not significantly changed ( p > 0.05, compared with NS, two sample t-Test, Figure 5I). The mean peak current density of *I*_A_ from NS-treated rats was 336.62 ± 70.31 pA/pF (n = 7), and the mean peak current density of *I*_A_ from CFA-treated rats was 288.58 ± 47.09 pA/pF (n = 7).

### CBS inhibitor AOAA reduces the H_2_S level and reverses hyperexcitability of TMJ neurons

**Figure 6:**
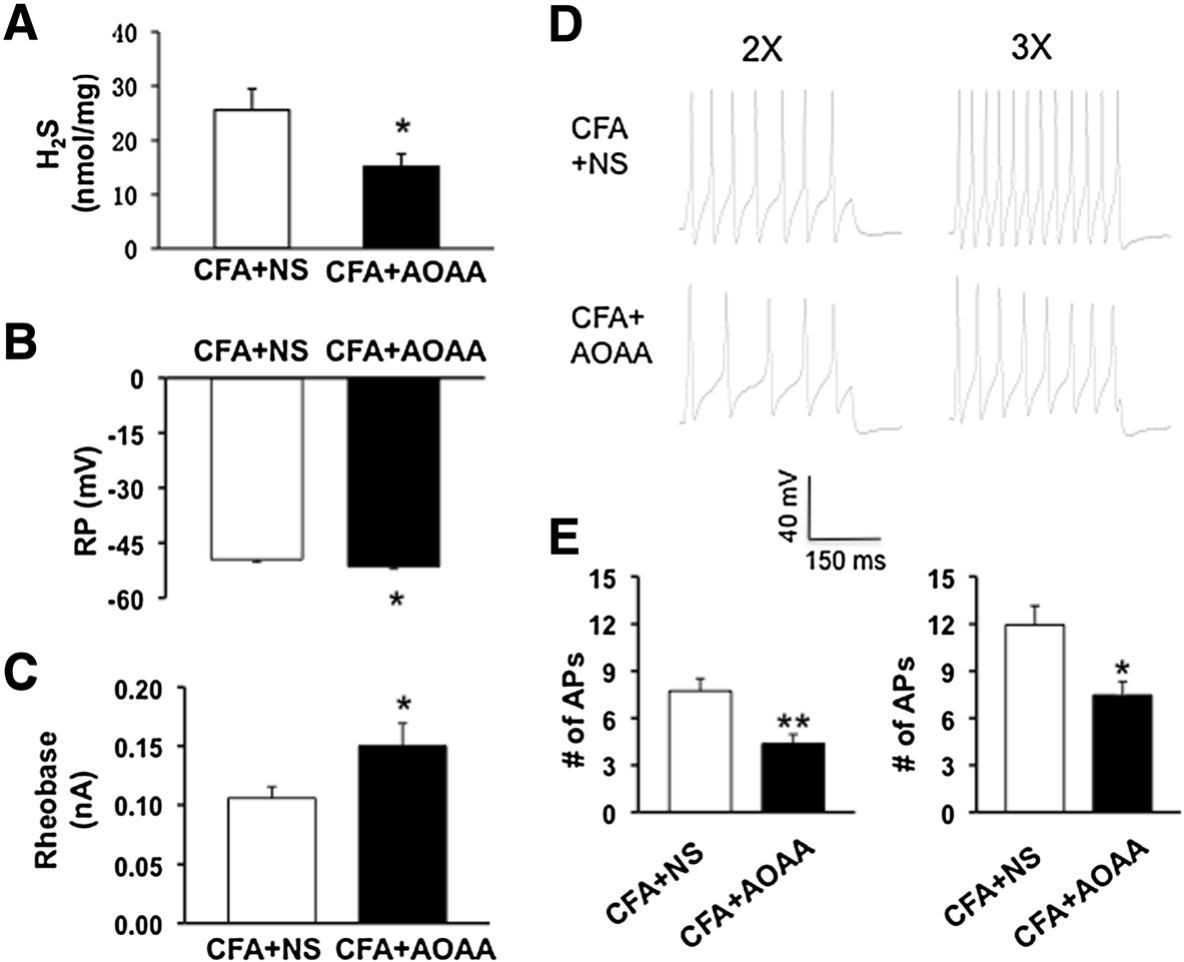
**CBS inhibitor AOAA reduces the H**_**2**_**S level and reverses neuronal hyperexcitability. (A)** Administration of AOAA (i.p., 9 mg/kg three times daily for consecutive 2 days) drastically reduced the level of H_2_S in TGs when compared with CFA rats (n = 9 rats for each group). **(B)** Administration of AOAA markedly hyperpolarized RP (CFA + AOAA, n = 20, CFA + NS, n = 20, *p < 0.05, compared with NS, two sample t-Test). **(C)** AOAA treatment significantly increased rheobase (CFA + AOAA, n = 20; NS, n = 20, * p < 0.05, compared with NS, Mann-Whitney test). **(D, E)** AOAA treatment also greatly reduced numbers of AP evoked by 2X (left) and 3X (right) rheobase current stimulation (CFA + AOAA, n = 20, CFA + NS, n = 20, *p < 0.05, **p < 0.01, Mann-Whitney test, two sample t-Test).

Since AOAA reversed the reduction in escape threshold in CFA rats, we next investigated whether AOAA treatment reduced the production of H_2_S in TG. As expected, administration of AOAA (i.p., 9 mg/kg three times daily for consecutive 2 days) drastically reduced the level of H_2_S in TGs when compared with CFA rats (Figure 6A, *p < 0.05, n = 9 rats for each group). We next determined whether administration of AOAA reversed hyperexcitability of DiI-labeled TMJ neurons from CFA injected rats. The resting membrane potentials (RPs) were -49.55 ± 0.59 mV (n = 20) and -51.60 ± 0.59 mV (n = 20) for NS and AOAA, respectively. AOAA treatment significantly hyperpolarized RPs of TG neurons from CFA injected rats (Figure 6B, *p *<* 0.05, two sample t-Test). Besides, AOAA treatment dramatically enhanced rheobase when compared with the NS-treated group (Figure 6C, *p < 0.05, Mann-Whitney test). The rheobase were 0.10 ± 0.01 nA (n = 20) and 0.15 ± 0.02 nA (n = 20) for NS and AOAA, respectively. AOAA treatment resulted in a significant reduction in the number of APs elicited in response to 2× and 3× rheobase current injections (Figures 6D and E, *p < 0.05, **p < 0.01, Mann-Whitney test, two sample t-Test). Figure 6C are representative voltage traces in response to 2× (left) and 3× (right) rheobase current injections after application of NS (top) or AOAA (bottom). The numbers of AP evoked by 2× rheobase current stimulation were 7.40±0.82 (n = 20) and 4.60±0.62 (n = 20) for NS- and AOAA-treated group, respectively. The numbers of AP evoked by 3× rheobase current stimulation were 11.40±1.27 (n = 20) and 7.85±0.98 (n = 20) for NS- and AOAA-treated group, respectively. This decrease in spike number was not due to a change in cell input resistance (NS vs. AOAA; 670.90±29.55 MΩ vs. 581.10±46.43 MΩ, p > 0.05, two sample t-Test).

**Figure 7:**
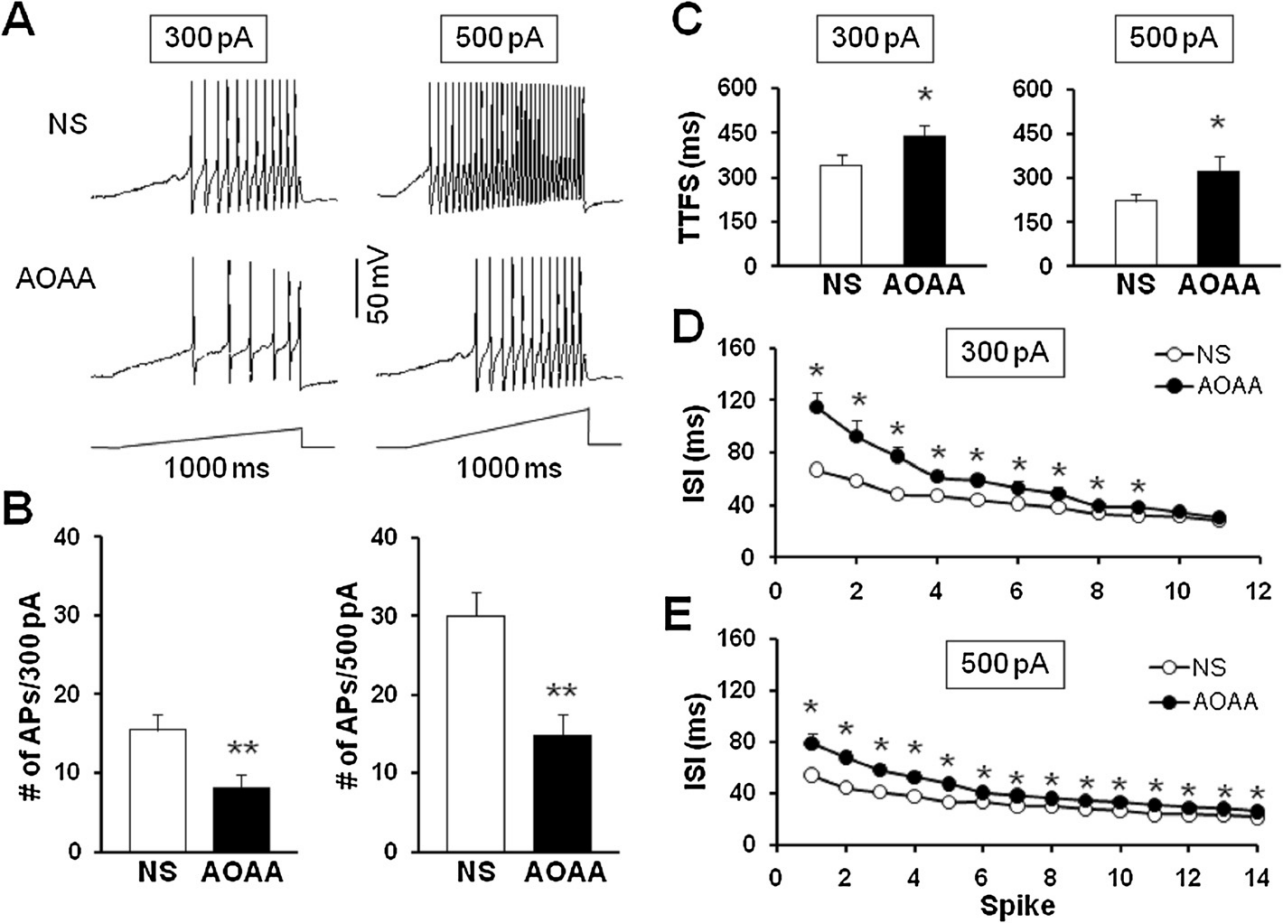
**CBS inhibitor AOAA reduces the number of action potentials evoked by ramp current stimulation. (A)** Examples of APs by 300 pA (left) and 500 pA (right) current stimulation from NS (top) and AOAA (bottom) treated rats. **(B)** Bar graph showed a significant decrease in numbers of APs evoked by 300 pA and 500 pA ramp stimulation in AOAA-treated rats (CFA + AOAA, n = 20, CFA + NS, n = 20, **p <0.01, compared with NS, Mann-Whitney test, two sample t-Test). **(C)** Bar graph representing the mean time to first spike (TTFS) in response to a 300 pA (left) or 500 pA (right) ramp current injection from NS or AOAA-treated rats (*p < 0.05, compared with NS, Mann-Whitney test). **(D)** Mean interspike interval (ISI) in a spike train of TMJ neurons responding to a 300 pA ramp current stimulation from NS- or AOAA-treated rats (*p < 0.05 compared with NS, Mann-Whitney test). **(E)** Mean ISI in a spike train of TMJ neurons responding to a 500 pA ramp current stimulation from NS- or AOAA-treated rats (*p < 0.05 compared with NS, Mann-Whitney test).

To further compare numbers of AP firing of TMJ neurons after AOAA treatment, we also used 1-second ramp current stimulation from 0 to 300 pA or 500 pA (Figure 7). Figure 7A shows the representative voltage traces in response to 300 pA (left) and 500 pA (right) ramp current stimulations 2 days after injection of NS (top) or AOAA (bottom). The average numbers of APs in NS-treated rats were 15.3±1.90 (n = 20) and 29.6±2.82 (*n = 20*) for 300 pA and 500 pA, respectively. In AOAA treated rats, the average numbers of APs were 8.1±1.73 (n = 20) and 15.65±2.65 (n = 20) for 300 pA and 500 pA, respectively. Injection of AOAA significantly decreased the number of APs evoked by 300 or 500 pA ramp current injection (Figures 7A and B, **p < 0.01, Mann-Whitney test, two sample t-Test). Again, this decrease in spike number was not due to a change in cell input resistance (NS vs. AOAA; 670.90±29.55 MΩ vs. 581.10±46.43 MΩ, p > 0.05, two sample t-Test). Furthermore, the time to first spike (TTFS) was significantly increased by AOAA treatment (NS vs. AOAA: 344.47±32.22 ms vs. 441.03±37.19 ms, *p < 0.05 at the 300 pA depolarization, Figure 7C left; 230.61±22.09 ms vs. 320.69±55.81 ms, *p < 0.05 at the 500 pA depolarization, Figure 7C right). A change in interspike interval (ISI) in response to a 300 pA and 500 pA current injection was seen both at the beginning of a train of spikes (after 1^th^ spike, NS vs. AOAA: 65.52±3.18 ms vs. 115.09±11.19 ms, *p < 0.05 at the 300 pA depolarization, Figure 7D; 53.97±2.85 ms vs. 78.96±8.01 ms, *p < 0.05 at the 500 pA depolarization, Figure 7E) and in the latter parts of the train (after 6^th^ spike, NS vs. AOAA: 41.01±2.42 ms vs. 53.44±5.12 ms; *p < 0.05 at the 300 pA depolarization, Mann-Whitney test, Figure 7D; 32.80±2.76 ms vs. 40.17±1.88 ms, *p < 0.05 at the 500 pA depolarization, Mann-Whitney test, Figure 7E), suggesting an effect of AOAA treatment on spike frequency adaptation of TMJ neurons.

### CBS inhibitor AOAA reverses voltage-gated potassium current of TG neurons

**Figure 8:**
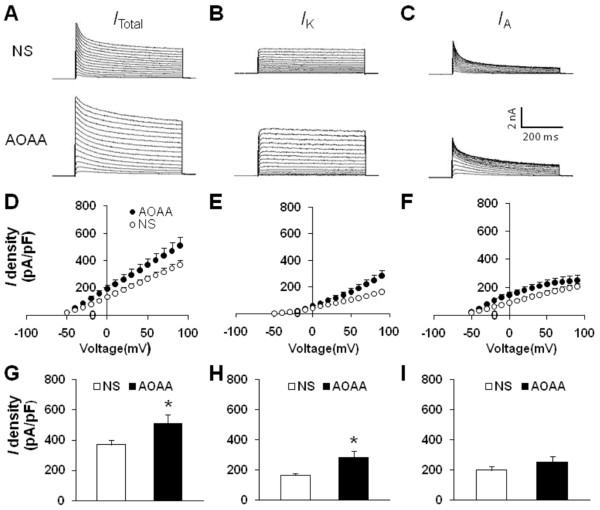
**CBS inhibitor AOAA increases voltage-gated potassium current.** Currents were measured at different holding potentials. For total voltage-gated potassium current (I_Total_), the membrane potential was held at -100 mV and voltage steps were from -50 to +90 mV with10-mV increments and 400 ms duration. For sustained K_V_ current (I_K_), the membrane potential was held at -50 mV and the voltage steps were the same as above. Currents generated by these two protocols were subtracted to produce I_A_. **(A)** Examples of total K_V_ currents recorded from NS (top) and AOAA-treated CFA rats (bottom). **(B)** Examples of I_K_ recorded from NS (top) and AOAA-treated CFA rats (bottom). **(C)** Examples of I_A_ recorded from NS (top) and AOAA-treated CFA rats (bottom). The peak I_Total_**(D)**, I_K_**(E)** and I_A_**(F)** versus voltages (I-V) were plotted from cells acutely dissociated from CFA rats treated with NS or AOAA. Bar graphs showed the mean peak I_Total_**(G)**, I_K_**(H)**, and I_K_**(I)** densities from NS and AOAA-treated CFA rats. The current density (pA/pF) was calculated by dividing the current amplitude by cell membrane capacitance. AOAA treatment caused a significant increase in I_Total_ (G, *p < 0.05, compared with NS, two sample t-Test). The I_K_ density was significantly increased (H, *p < 0.05, compared with NS, two sample t-Test) while the I_A_ was not altered significantly after AOAA treatment in CFA rats **(I)**.

Since AOAA reversed hyperexcitability of TMJ neurons in CFA rats, we next investigated whether AOAA suppressed current density of K_V_ current in DiI labeled TG neurons. Rats were divided into two groups: AOAA group treated with AOAA (9 mg/kg) and NS group treated with the same volume of normal saline. The mean peak current density of total voltage-gated potassium current from AOAA-treated rats was 508.91 ± 61.75 pA/pF (n = 8), and the mean peak current density of total voltage-gated potassium current from NS-treated rats was 369.81 ± 31.84 pA/pF (n = 10). AOAA treatment significantly reversed the reduction of peak amplitude of *I*_Total_ (Figures 8A, D &G, *p < 0.05, compared with NS, two sample t-Test). As expected, AOAA treatment remarkably increased the mean peak current density of *I*_K_ (*p < 0.05, compared with NS, two sample t-Test, Figures 8B, E&H). The mean peak current density of *I*_K_ from AOAA-treated rats was 283.74 ± 42.38 pA/pF (n = 8), and the mean peak current density of *I*_K_ from NS-treated rats was 163.66 ± 11.79 pA/pF (n = 10). However, *I*_A_ density was not significantly changed (p > 0.05, compared with NS, two sample t-Test, Figures 8C, F&I). The mean peak current density of *I*_A_ from AOAA-treated rats was 251.17 ± 38.39 pA/pF (n = 8), and the mean peak current density of *I*_A_ from NS-treated rats was 202.99 ± 24.48 pA/pF (n = 10).

## Discussion

The present study was designed to determine the effects of CBS-H_2_S signaling on nociceptive processing in trigeminal ganglion cells innervating the TMJ of rats under pathophysiological conditions. We first examined the role of CBS-H_2_S signaling on excitability of TG neurons. Injection of a CBS inhibitor reduced excitability of TG neurons in rats with TMJ inflammation induced by CFA injection. The AOAA treatment appears to modulate the response of a TG neuron to suprathreshold inputs and therefore have an important role in determining the output of the neuron. Specifically, injection of AOAA led to a significant decrease in spiking activity in response to current injection. AOAA treatment reduced the number of action potentials evoked at any given current injection, enhanced threshold of excitation, increased latency to first spike and the interspike interval (ISI) throughout the spike train. Importantly, the reduced escape threshold produced by CFA injection was antagonized by the presence of AOAA, confirming that these effects are likely mediated through CBS signaling. Collectively, these data suggest that CBS-H_2_S signaling plays a crucial role in inflammatory pain in TG cells and most likely acts to modulate TG neuronal excitability.

A unique feature of this study is the local *in vivo* use of AOAA. AOAA, as a potent inhibitor for CBS, has been widely used in many fields [32]. However, AOAA could produce non-specific effects such as a blunted response to hypoxia when it is used systematically or in a large dose [33]. Therefore, we chose subcutaneous injection of AOAA to avoid possible side effects produced by AOAA. To exclude possible role of AOAA on rat motor coordination/function, the Rota-Rod test was performed in the present study. No significant difference (p > 0.05) was observed in the time that animals remained on the rota-rod at 15 rpm before and after AOAA treatment (data not shown), indicating that AOAA-induced analgesic effect is not due to the reduced motor function. Subcutaneous injection of AOAA significantly attenuated the pain behavior in CFA rats, in a dose- and time-dependent manner. No significant effect was seen in control animals, suggesting that this was not a non-specific analgesic effect. This also suggests that the role of CBS pathway in signaling TMJ information may not be as important in health as in the sensitized pathophysiological state. Since cystathionine-γ-lyase (CSE), another endogenous H_2_S producing enzyme, was not altered in terms of expression after CFA injection, we focused our study on the effect of CBS. If H_2_S generated endogenously contribute to the development of mechanical hyperalgesia in CFA-injected animals, application of exogenous H_2_S to healthy rats should mimic the effects of CFA. Therefore, we applied L-Cys, an endogenous substrate for CBS to generate H_2_S, to healthy rats and assessed behavioral responses. Addition of L-Cys mimics the CBS production of H_2_S. Together with our previous report [15], these data suggest that CBS-H_2_S signaling plays a crucial role in inflammatory pain in TMJ.

Another important change is the inflammation-induced upregulation of CBS expression observed in TGs. CFA injection upregulated CBS expression at both protein and mRNA levels. This is similar to those observed in rat hindpaw [13], colon [34] and gastric [14] afferents. That such a change has been observed in afferents innervating three different tissue types inflamed with different stimuli suggests that an increase in CBS expression may be a general response to inflammatory injury. However, expression of CBS was not altered in the rat model of sciatic nerve injury model [13] and bone cancer pain model (personal unpublished data), suggesting a disease-specific upregulation of CBS expression. The basis for such an increase is unclear but may be associated with epigenetic mechanisms such as DNA demethylation [13] or regulated by transcriptional factors such as nuclear factor kappa B [14] under pathophysiological conditions. The detailed molecular mechanisms underlying the upregulation of CBS gene expression in TMJ afferents need to be further investigated.

Much of the published data to date suggest that H_2_S, formed by two enzymes CBS and CSE, regulates key neuronal functions. These include induction of long-term potentiation and modulation of NMDA receptor currents in the hippocampus under physiological conditions [35,36]. Recently, H_2_S has also been reported to enhance excitability of stomach [34], colon [11], and hindpaw [13] innervating dorsal root ganglion neurons *in vitro*. In present study, we provide direct evidence for CBS signaling involved in hypersensitivity of TMJ innervating TG neurons in the setting of TMJ inflammation. We first confirmed that TMJ inflammation enhanced neuronal excitability. This conclusion is based on several findings shown in Figures 3 and 4. Firstly, TMJ neurons from animals with inflammation displayed marked depolarization of resting membrane potential. Secondly, these neurons exhibited lower current thresholds for initiating an AP compared with controls. Thirdly, these neurons had enhanced firing frequencies in response to a standardized stimulation compared with controls. Finally, TMJ neurons from CFA-treated rats had enhanced firing frequencies and reduced latency to first spike and interspike interval (ISI) in response to a ramp current stimulation when compared with controls. This is similar to that reported by Flake et al. [16]. Of note is that such reduced ISI might be influenced by the net inward current (i.e. the reduced *I*_K_) during the ISI and thus influence firing frequency. Interestingly, both the pattern of inflammation-induced excitability changes and the associated changes in passive properties, properties of the action potential waveform, or specific ion channels varies from study to study. For example, the inflammation-induced increase in cell body size was observed in TMJ [16], bladder [37] and gastric [38] afferents as well. That such a change has been observed in afferents innervating three different tissue types inflamed with different stimuli suggests that an increase in cell body capacitance seems to be a general response to inflammatory tissue injury. However, no significant change in cell body size was observed in pancreas afferents in a rat model of chronic pancreatitis [39]. It is possible that differences between these studies as well as the present study reflect differences in experimental methods (i.e., type of inflammation, timing between induction of inflammation and study of neurons, species and sex of animals). Nevertheless, our data suggest that CFA-induced TMJ inflammation enhanced neuronal excitability, which is presumably mediated by CBS-H_2_S signaling. We then provided direct evidence to support our hypothesis. Local administration of CBS inhibitor AOAA reversed the enhanced excitability of TMJ neurons as evidenced by an increase in rheobase, a reduction in the numbers of evoked action potentials, and hyperpolarization of resting membrane potentials. These changes in electrophysiological properties of TMJ neuron support the changes in pain behaviors after AOAA treatment. Together with our previous report that H_2_S enhanced excitability of TG neurons [15], the present study further indicates that H_2_S modulates membrane properties of rat TG neurons under pathophysiological conditions.

The ionic basis for the reduced excitability by AOAA remains unknown but may reflect an alteration in the biophysical properties and/or expression of one or more ion channel(s) such as voltage-gated sodium, potassium and calcium channels [7,40]. H_2_S has been reported to modulate activities of different channels such as K_ATP_ currents [12], T-type calcium [8] and sodium channel current [13] of DRG neurons, and the sustained potassium current of TG neurons [15]. Since we have previously demonstrated that CBS was co-localized with K_V_1.1 and K_V_1.4 and that H_2_S donor NaHS suppressed the *I*_K_ current density [15], we continued to examine the effect of AOAA on K_V_ currents in present study. AOAA treatment significantly enhanced the *I*_K_ current density, supporting the hypothesis that *I*_K_ plays an important role in TMJ inflammatory pain. Of note is that contributions from other ion channels cannot be excluded in the present study. Further researches into detailed mechanisms of TMJ pain are definitely necessary.

In conclusion, the roles of CBS-H_2_S signaling in nervous system function are still being deciphered but it is becoming rapidly clear that CBS-H_2_S signaling can have profound influences on brain and cellular activity. The data presented here demonstrate yet another locus for modulation of activity at peripheral nervous system by CBS-H_2_S signaling. As we continue to uncover the wide-ranging effects of CBS-H_2_S activation, we will hopefully reveal potentially new strategies for therapeutic interventions in a wide array of common diseases such as chronic pain.

## Competing interests

The authors declare that they have no competing interests.

## Authors’ contributions

XMiao and XMeng performed experiments, analyzed data, prepared figures and drafted the manuscript. GW performed experiments, analyzed data. ZJ and HHZ performed experiments. SH analyzed data, prepared figures. GYX designed and supervised the experiments, and edited the manuscript. All authors read and approved the final manuscript.
